# Sequencing and Characterization of Striped Venus Transcriptome Expand Resources for Clam Fishery Genetics

**DOI:** 10.1371/journal.pone.0044185

**Published:** 2012-09-18

**Authors:** Alessandro Coppe, Stefania Bortoluzzi, Giulia Murari, Ilaria Anna Maria Marino, Lorenzo Zane, Chiara Papetti

**Affiliations:** Department of Biology, University of Padova - via G. Colombo, Padova, Italy; Auburn University, United States of America

## Abstract

**Background:**

The striped venus *Chamelea gallina* clam fishery is among the oldest and the largest in the Mediterranean Sea, particularly in the inshore waters of northern Adriatic Sea. The high fishing pressure has lead to a strong stock abundance decline, enhanced by several irregular mortality events. The nearly complete lack of molecular characterization limits the available genetic resources for *C. gallina*. We achieved the first transcriptome of this species with the aim of identifying an informative set of expressed genes, potential markers to assess genetic structure of natural populations and molecular resources for pathogenic contamination detection.

**Methodology/Principal Findings:**

The 454-pyrosequencing of a normalized cDNA library of a pool *C. gallina* adult individuals yielded 298,494 raw reads. Different steps of reads assembly and filtering produced 36,422 contigs of high quality, one half of which (18,196) were annotated by similarity. A total of 111 microsatellites and 20,377 putative SNPs were identified. A panel of 13 polymorphic transcript-linked microsatellites was developed and their variability assessed in 12 individuals. Remarkably, a scan to search for contamination sequences of infectious origin indicated the presence of several Vibrionales species reported to be among the most frequent clam pathogen's species. Results reported in this study were included in a dedicated database available at http://compgen.bio.unipd.it/chameleabase.

**Conclusions/Significance:**

This study represents the first attempt to sequence and *de novo* annotate the transcriptome of the clam *C. gallina*. The availability of this transcriptome opens new perspectives in the study of biochemical and physiological role of gene products and their responses to large and small-scale environmental stress in *C. gallina*, with high throughput experiments such as custom microarray or targeted re-sequencing. Molecular markers, such as the already optimized EST-linked microsatellites and the discovered SNPs will be useful to estimate effects of demographic processes and to detect minute levels of population structuring.

## Introduction

The class Bivalvia is characterised by a substantial economic interest chiefly because many species are target of commercial fishery and aquaculture [1]. Despite the high number of clams species, only about two dozen are fished in commercial quantities. Bivalves contribute with a small percentage (∼2%) to global capture fishery landings but their generally high price compensates for the smaller landed weight when compared with other categories of fish, crustaceans and molluscs [1]. Bivalve species have also important roles on highly variable coastal ecosystems and environments, for instance accumulating toxic substances, including heavy metals, by filter-feeding. Strong changes in the environmental conditions of these systems and an increased fishing pressure, may affect bivalve production and have a detrimental impact on the role that these organisms play in coastal ecosystems by challenging their ability to cope with multiple stresses such as temperature variability or alien species competition [2], [3].

The striped venus *Chamelea gallina* (Linnaeus, 1758; Mollusca, Family Veneridae) is a marine bivalve distributed throughout the Mediterranean, in the Black Sea, along the Portuguese coast and in a few localities of the northern Atlantic [4], [5], [6]. Besides being one of the oldest [7], with ∼100,000 t annual landings, the commercial fishery of this species is also among the largest clam fishery in the Mediterranean Sea, particularly in the inshore waters of northern Adriatic Sea [8]. The high fishing pressure has lead to a strong decline in stock abundance [9] further enhanced by several irregular mortality events. Since these conditions could lead to rapid evolutionary changes, with consequences for the genetic structure and implications for stock management [10], future investigations should define whether this species has sufficient genetic potential to adapt to the predicted changes in the abiotic characteristics of oceans and to cope with the strong fishing pressure. Next Generation Sequencing (NGS) technology and its downstream applications provide today rich genomic resources to address these issues. In particular, the transcriptome sequencing represents an effective way of looking into the genome focusing only on transcribed regions [11]. Moreover, deep sequencing of transcriptomes is able to rapidly bring non-model organisms into the post-genomic era, also those nearly completely uncharacterized from a molecular point of view [12], [13], [14] such as *C. gallina* for which only 12 nucleotide species-specific sequences were available in GenBank database, so far. In this study, we applied the 454 pyrosequencing technology to obtain the first *C. gallina* normalized transcriptome library. Starting from raw sequencing data, the transcriptome of the striped venus was reconstructed, annotated and analyzed. Results obtained with this approach were included in a dedicated database. In addition, we performed a transcriptome scan to search for pathogenic contamination in our sample. Considering the high risk of human disease transmission via molluscs consumption, the characterization of new, potentially pathogenic sequences in economically relevant species could provide more accurate tools for commercial products screening. Moreover, the availability of *C. gallina* transcriptome opens new perspectives in the study of biochemical and physiological role of gene products and their responses to large and small-scale environmental stress.

## Results and Discussion

### Transcriptome assembly and contigs quality-based selection

A cDNA sample obtained from a RNA pool of four *C. gallina* adult individuals was used to produce a normalized library. This library was sequenced using half a plate of Roche 454 GS FLX platform (Life Sciences, Branford, CT, USA). This single sequencing run produced 298,494 raw reads, with an average sequence length of 210 nucleotides (nt). In the pre-processing phase short reads (<60 bases) were removed and low-quality sequence regions (<30 Phred quality) were trimmed, obtaining 298,369 reads. Thus, 99.96% of the raw reads contained potentially useful sequence data. Raw sequencing data were submitted to Sequence Read Archive (SRA) SRA052281.

Using MIRA assembler, trimmed reads were assembled into contigs by two successive assembly runs. The first assembly run of 298,369 reads produced 41,630 contigs from 202,125 assembled reads (68%). The average length of these contigs was 335 nt.

Due to the heuristic nature of the assembly process and previous reports of some degree of redundancy (i.e. different contig sequences belonging to the same transcript region) in sets of transcriptome contigs assembled with different methods [15], a second run of assembly was conducted as done for previous studies [12], [14]. The second assembly step was run using the set of reads discarded by the first run together with contigs from first run. Re-assembly produced 3,082 meta-contigs including 4,962 contigs and 2,514 reads, whereas 36,668 contigs remained unchanged. The stringent criteria applied for the second assembly run allowed to reduce contig sequences redundancy, to produce a slight increment in the number of assembled reads and sequence coverage, and to increase contig length. In total, 69% of original read sequence information was used to obtain 39,750 contigs, with an average contig length of 351 nt.

**Figure 1:**
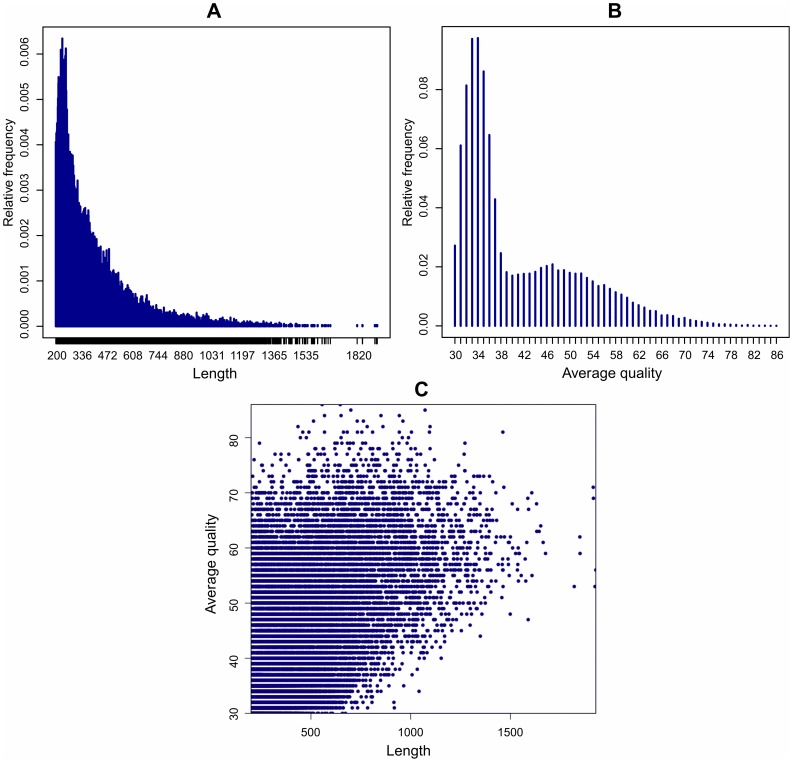
Length and quality of *Chamelea gallina* transcriptome contigs. Panel histograms report the contig's length (a) and average quality (b) distribution, while panel c shows the relationship between length and quality.

Further contig filtering, by length and quality, retained only contigs at least 200 nt long with an average sequence quality of at least 30, corresponding to a mean probability of wrong base calling of less than 10^−3^. The final set of 36,422 contig sequences, considered of adequate high quality, *bona fide* represent a portion of *C. gallina* transcriptome. Mean and median contig length were respectively 352 and 289 nt (Fig. 1a). Mean and median values of average contig quality were 39.8 and 35,4, respectively (Fig. 1b). The scatterplot in Fig. 1c depicts the relationship between length and average quality of transcriptome contigs.

### Functional annotation by similarity

*De novo* functional annotation of *C. gallina* transcriptome was obtained by a multistep procedure, starting with similarity search against main nucleotide sequence databases, used for transferring functional information, by sequence similarity, from a species to another, with the final aim to infer possible function of proteins encoded by newly sequenced transcripts.

#### BLAST against protein and nucletide sequence databases

The set of 36,422 contig sequences was compared against the nr protein database with BLASTX. This step allowed the identification of significant similarity with known proteins for 8,601 contigs (23.6%, e-values distribution for nr: 1^st^ quartile 0.000e+00, median 0.000e+00, 3^rd^ quartile 1.085e−07). The fraction of reconstructed transcriptome with nr BLAST was approximately matching the 26% of the transcripts as observed for other molluscs [11], [16], [17], [18].

The majority of contig sequences (27,821; 76.4%) were not associated to any nr BLAST hits, i.e. to known proteins. The lack of significant similarity with known proteins for the three quarters of contigs could be explained by several reasons, as low conservation of part of the protein coding mRNAs, of long non-coding RNAs, if any, and relative shortness of sequences available from closely related mollusc species in biological databases. Part of transcriptome can be non full-length (a few contigs may represent only a transcript fragment). Indeed, a comparison between sequences with and without nr BLAST hits showed differences in length and quality: annotated sequences were longer (482.3 nt on the average) and of higher quality (45.8) than non-annotated sequences (359.4 nt on the average and 39.9). Considering alignment coverage between each query (contigs) and the subject sequences (known proteins), aligned regions covered on average 17.6% of contigs length and 60.1% of subject sequence length. In terms of sequence completeness, the contigs can be strictly defined full-length if they include the complete 5′ and 3′UTRs. Broadly adopted definition considers a sequence as full-length when it contains the complete coding sequence (CDS). Among 8,601 contigs with significant protein hits, 811 were aligned with the subject sequence for at least 70% of the protein length, while 1,652 covered the 50% of the subject sequence.

Moreover, a considerable transcriptome portion can comprise non-coding sequences, namely mRNAs non-coding regions (5′ and 3′ UTRs) as well as other long and moderately long non-coding RNAs. Recent experimental studies have demonstrated that eukaryotic genomes are pervasively transcribed and extensive sequencing projects are progressively identifying new categories of non-coding RNAs [19]. In this view, it is interesting to notice that current estimates of the number of long non-coding RNAs (lncRNAs) in the human genome ranges from 5,000 to 20,000, with evidences about poor conservation of lncRNAs across species, also at a relatively small phylogenetic distance (e.g. intra-amniotes comparison, [20]).

Transcript sequences were also compared to nt database (nucleotide sequences, including GenBank, EMBL, and DDBJ databases but excluding bulk divisions (gss, sts, pat, est, and htg) and wgs entries), using BLASTN. We identified significant similarity with nt hits for 13,314 transcripts (37%, e-values distribution for nt: 1^st^ quartile 7.840e−08, median 4.077e−07, 3^rd^ quartile 1.909e−06).

**Figure 2:**
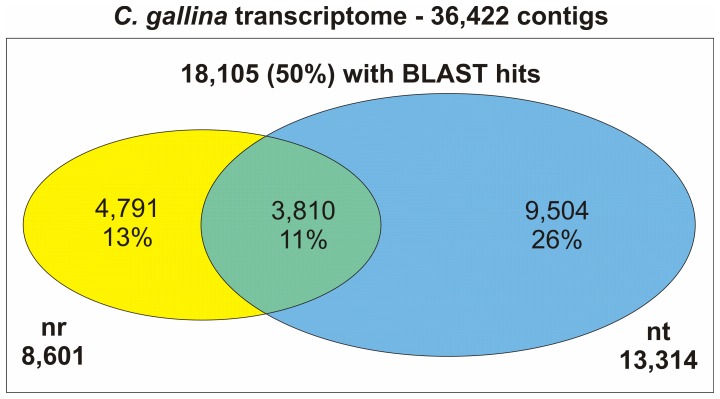
Set of annotated contigs of *Chamelea gallina* trascriptome with nucleotide and protein BLAST hits. The Venn diagram reports the intersection between number of contigs with BLAST hits in nr protein and nt nucleotide databases.

When merging results of the two BLAST searches (Fig. 2), 9,504 contigs (26%) have only nucleotide hits, while 3,810 contigs (11%) showed significant BLAST hits in both protein and nucleotide databases. In this way, we have been able to annotate 50% of *C. gallina* transcriptome, since 18,105 contigs resulted to be similar to at least one known bio-sequence available in nr or nt databases.

Additional search in Pfam/Rfam databases to retrieve matches with known domains and/or non-coding RNAs (Table S2) did not provide a major improvement to the annotation, since only 91 new contigs were successfully annotated. Final number of annotated contigs was 18,196.

#### Functional annotation with Blast2GO

**Figure 3:**
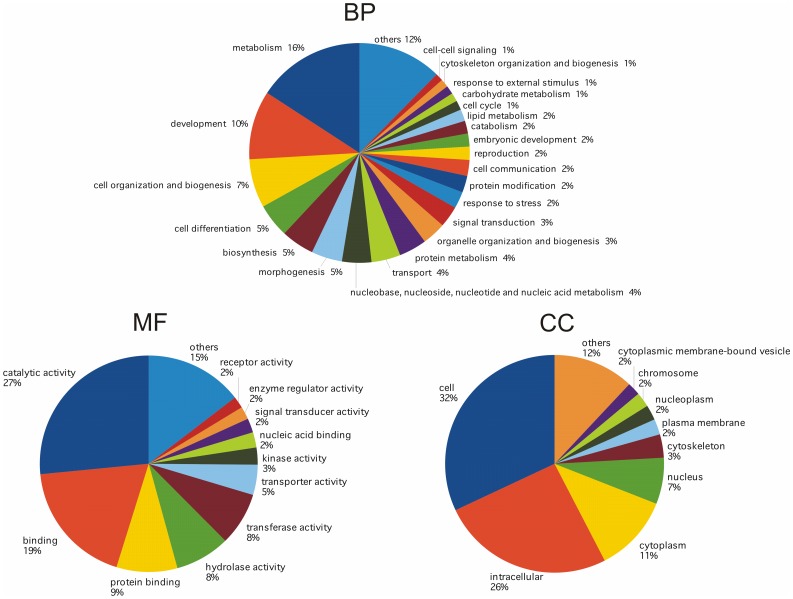
*Chamelea gallina* transcriptome functional annotation based on Blast2GO analysis. Functional annotation results indicate the relative amount of each category of contigs with protein hits. The results are summarized as follows: Biological Process (BP), Molecular Function (MF) and Cellular Component (CC).

Among 8,601 contigs with nr BLASTX hits, 5,032 (58.5%) were associated to one or more 3,577 unique GO terms, for a total of 32,416 term occurrences (Fig. 3a). Using the web tool CateGOrizer, the 32,614 GO-terms were grouped into a total of 124 GO-Slim terms (Fig. 3b), which included biological process (57.5%), molecular function (23.0%) and cellular component (19.5%) ontologies. Belonging to at least one of the 11 ‘GO-slim2’ classes, 3,577 unique terms were found, while 395 “odd” terms did not belong to any classes. Among biological processes, cellular, regulatory and development processes represented 95% of the total, although other key processes like growth, reproduction or death were also present. Among molecular function terms, “Binding” and “Catalytic activity” represented about 38% and 27%, respectively.

Despite the relative ease of achievement of an ultra-high throughput sequencing of cDNA libraries, molluscs resources are still lacking and in particular, for Bivalvia, cDNA clones sequencing and microarray hybridization are still a solution of choice. The present transcriptome represents the first step towards a complete sequencing of the striped venus genome which will allow to get also low expression genes usually less represented in a normalized cDNA library. Moreover, the sequencing of different developmental stages and not only adults will allow to retrieve genes specifically expressed for each stage and to complete the isolation of the total available messengers. An additional challenging issue is the increase of percentage of clam transcripts that can be matched against a known protein-coding gene (nr BLAST hits). The phylogenetic distance of molluscs from other metazoan model species (e.g. *Drosophila melanogaster*, *Caenorhabditis elegans*, *Danio rerio*, *Mus musculus* and *Homo sapiens*) has greatly reduced the power of functional annotation comparative approach. We expect this limitation to be quickly overcome by the acquisition of new resources. In this sense, a substantial step forward has been recently achieved thanks to the efforts of the Oyster Genome Consortium with the completion of the Pacific oyster, *Crassostrea gigas* (Mollusca, Bivalvia) genome sequencing (WGS BioProject), published on 15 July 2011 [21]. The economic importance of the Pacific oyster with a worldwide aquaculture production of over 4 million metric tons, has fuelled a large number of studies on the ecology, physiology, immunology, and genetics of this species populations. The recent genome sequencing fulfilment of *C. gigas* has opened new chances towards the production of targeted gene knock down individuals [22], [23]. In addition, the forthcoming accomplishment of *de novo* sequencing and annotation of the two gastropods *Aplysia califonica* and *Lottia gigantea* complete genomes and the recently published *Meretrix meretrix* (Bivalvia, [24]), *Laternula eliptica* [Bivalvia, 16], *Solemya velum* and *Nucula nitidosa* (Bivalvia, [25]), *Lymnaea stagnalis* (Gasteropoda, [26]) and *Crepidula fornicate* (Gasteropoda, [27]) transcriptomes and EST resources will provide a precious instrument for the analysis of mollusc species sequences.

### Gene-associated molecular markers

Novel microsatellite (Simple Sequence Repeats, SSR) and Single Nucleotide Polymorphisms (SNPs) were detected after specific search within *C. gallina* transcripts.

In total, 111 SSRs were identified in 105 out of 36,422 transcript sequences (Table 1). The most frequent repeat motifs were trinucleotides, which accounted for 38.7% of all SSRs, followed by tetranucleotides (31.5%), dinucleotides (23.4%), pentanucleotides (4.6%), and hexanucleotides (1.8%) (Table 2). Within the dinucleotides, the AT motifs (8 and 9 repeats) represented the most abundant and corresponded to approximately the 26.9% of the whole category. The most common motif among trinucleotide repeats was AAC (14.3%), followed by CAA, GAT, TCA and TTG (11.9%), whereas the most abundant tetranucleotide motifs were ACAG and TGTA (9.1%). All pentanucleotide motif were equally frequent.

**Table 1:** *Chamelea gallina* SSR mining results.

**Total number of sequences examined**	36,422
**Total size of examined sequences**	14,146,368
**Total number of identified SSRs**	111
**Number of SSR containing sequences**	105
**Number of sequences containing more than one SSR**	6
**Number of composite SSRs**	6

The table reports the main results provided by MISA for SSR detection.

**Table 2:** Summary of SSR types and frequency in *Chamelea gallina* transcriptome.

Type	#	% of contigs containing at least one SSR
**Di-nucleotides**	26	23.4
**Tri-nucleotides**	43	38.7
**Tetra-nucleotides**	35	31.5
**Penta-nucleotides**	5	4.6
**Hexa-nucleotides**	2	1.8
**Total**	111	100.0

We selected 46 loci for primer design. Genomic DNA from 12 *C. gallina* individuals was used to test amplification success and loci variability. Among the 46 primer pairs, 9 did not provide any amplified fragment, 8 showed a longer unexpected fragment length (by agarose gel sizing), possibly suggesting the presence of an intron interrupting sequenced regions, and 4 displayed a multi-band pattern. Of the 25 left pairs, all were polymorphic in the initial screening via agarose gel, but 11 did not consistently produce any PCR fragment for several genomic DNA samples tested, suggesting the hypothesis of null alleles or large allele drop out affecting these loci. The final markers panel entailed one monomorphic and 13 polymorphic loci (Table 3). The allele number per locus ranged from 2 to 14, with an average value of 5.2 (Standard Deviation SD ±3.6). Mean observed and expected heterozygosities were 0.36 (SD ±0.23) and 0.61 (SD ±0.25) (Table 3). Seven loci out of 13 were in Hardy-Weinberg Equilibrium (calculated on 12 individuals, a limited number of samples). Hardy-Weinberg disequilibrium was generally due to homozygosity excess. Since the population sample was very small, HWE results may be due to single locus stochasticity. It could be also expected that enlarging the sample size would allow to identify additional alleles for the monomorphic locus.

**Table 3:** Variability assessment of 14 SSR loci of *Chamelea gallina*.

Locus name	Repeat content	Primers (5′-3′)	Fluorescent label	Ta (°C)	Size range (bp)	Allelic range (repeats)	Na	Ho	He	pHWE^a^
260	(TA)_6_	F:TGCTCATAACGGCAAAGTACA/R:TGGAGTTGACGATGTAACCCA	FAM	54	92–96	2	3	0.7500	0.5399	0.2826
1088	(TA)_6_	F:ATCGGAAGACGACGATGCATG/R:GCAACTTCCGACATAATGGGA	FAM	56	143–166	12	5	0.1667	0.7355	**0.0001** *
1243	(TGTT)_5_	F:AGTTATGGAACAGGCATAGCA/R:GTAGAAGGAAGCCAGACTCAC	VIC	54	107–118	6	4	0.2500	0.5870	**0.0070** *
3263	(AT)_6_	F:TCGCACGATTTACTCTTCCGT/R:ATGTTGTTGTCTCGCTAGCCA	VIC	57	103–110	5	6	0.4167	0.8261	**0.0008** *
9969	(TCT)_6_	F:TGGAACCAAAATTCACAGGTGA/R:TGCATTCTCATTACTGTCCTCA	NED	56	132–135	1	2	0.0000	0.2899	**0.0063** *
10343	(GTT)_7_	F:AGCAAATTGGCACTTGTCAGC/R:AACGTTACACCTGTGATTCCT	VIC	55	244–250	2	3	0.2500	0.4529	0.0905
18241	(TCA)_6_	F:ACTAGGTGTTATCCAGCCATCA/R:GAGTTGGGAGAAGGGTGACAC	PET	56	116–134	6	6	0.5833	0.5543	0.8236
20070	(AG)_6_	F:AGCAGTTCCTTGTCAATACCA/R:TTAGTGGCGTCGCTATTTTGT	VIC	54	172–184	6	7	0.5000	0.8442	**0.0068** *
20447	(GT)_6_	F:TGCCCTTTAGCACATTGAGCT/R:GTAAGGCCCAAAGCGGTGTGT	PET	56	230–232	1	2	0.1667	0.1594	1.0000
20467	(ATTA)_5_	F:GGGGACCAGAAACTATTTGGCT/R:CGTCCATCCTAACTGTAACACT	NED	58	96–109	7	6	0.2500	0.7536	**0.0001** *
26069	(ATG)_6_	F:CTGAACAACTGTCTGCATGAC/R:CGCCCAAAGAAGTCTTGATGA	VIC	57	184–190	2	3	0.1667	0.3043	0.0885
33835	(ATTG)_10_	F:CATGATTATGGACCCCTTCAC/R:CCGACTATATCAGACGTTCAGG	FAM	55	247–327	44	14	0.5833	0.9275	**0.0005** *
41629	(AT)_6_	F:TGCCTTTGTTCTGAAAGCAGT/R:ACCTTGAGCAAGTTAGCTGGCT	NED	55	241–286	25	11	0.6667	0.9058	**0.0072** *
41630	(TA)_6_	F:CGCTCTCTACCAATGCATCCA/R:TGCTCCTTAGCATCACAGAAC	PET	56	178	-	1	-	-	-
Mean (±SD)							5.2142 (±3.6199)	0.3654 (±0.2320)	0.6062 (±0.2513)	-

Variability, expressed in terms of number of different alleles, was assessed on 12 individuals collected in Chioggia in 2010 (off Venice lagoon, Italy). The table reports the name of each locus, taken from the contig number, the repeat content, the forward (F) and reverse (R) primer sequences, the fluorescent label, the annealing temperature (Ta) of PCR amplification, the size range of amplified fragments in bp, the allelic range in repeats, the number of alleles (Na) detected and the Hardy-Weinberg probability (pHWE). Significant p-values in bold (α = 0.05). Mean values for allele number, observed and expected heterozygosity are reported in the last row. Standard Deviation is reported in brackets (± SD).

* Loci putatively affected by null alleles following MICRO-CHECKER 2.2.3. [51].

a p-values were calculated based on a limited number of individuals (n = 12).

High quality putative SNPs were selected with FreeBayes software [28]. We identified 20,377 trustable SNPs (phred quality score >10) out of 6,267 contigs (17%). These putative SNPs included 12,281 transitions and 8,096 transversions (Table 4) and the overall frequency of all SNPs types found, excluding indels, was one per 697 bp. Among detected SNPs, 5,656 where located in 1,578 annotated contigs.

**Table 4:** Putative SNPs identified from *Chamelea gallina* transcriptome database.

SNP type	Number
*Transitions*	12,281
A-G	6,389
C-T	5,892
*Transversions*	8,096
A-C	1,928
A-T	3,194
C-G	1,013
G-T	1,951
*Total*	20,377

More than 20,000 SNPs were identified out of 36,422 contigs and meta-contigs.

### Identification of pathogenic sequences in *Chamelea gallina* transcriptome

Bivalve molluscs are especially prone to act as transmitters of human disease-causing pathogens [29]. The waters they inhabit are often exposed to contamination by faecal matter from sewer drains or from infected individuals. It has been widely shown that not only the consumption of contaminated seafood can cause diseases in humans, but this can likely represent a possible cause of molluscs high mortality events [29]. The pathogenic species more often reported in *C. gallina* are *Marteilia refringens* (Rhizaria, Canadian Food Inspection Agency, http://www.inspection.gc.ca), *Cryptosporidium spp.* (Apicomplexa, [30], [31]), *Vibrio tapetis* [Order Vibrionales, Brown Ring Desease, 1] and *Perkinsus olseni* (Alveolata, [32]). Molecular genotyping is of key importance for unequivocal identification of these species, to define the environmental or animal origin of infection and to study the epidemiology and transmission patterns.

Starting from this information, we blasted *C. gallina* contig database against the taxonomic groups of Rhizaria, Alveolata (including Apicomplexa), and order Vibrionales. We retained only matches longer than 100 nt with over 95% similarity and e-value smaller than 1e-3. Filtered results indicated the presence of Vibrionales sequences in the clam transcriptome sample (Table S1), with a maximum e-value of 2e−85. Vibrionales species are reported to be commonly found in *C. gallina* individuals [33] indicating that our BLAST outputs represent a biologically meaningful result. Together with additional available resources for other clam species (*Ruditapes philippinarum*, [34]), the investigation of microbial, bacterial and viral transcripts in *C. gallina* contig dataset provides new and possibly more reliable molecular tools to investigate the role of parasites and pathogenic species in the striped venus mortality events reported in the coastal waters of northern and central Adriatic.

### ChameleaBase: the *Chamelea gallina* transcriptome database

A specific database for *C. gallina* transcriptome has been implemented using MySQL and Django web facilities and it is freely available at http://compgen.bio.unipd.it/chameleabase.

**Figure 4:**
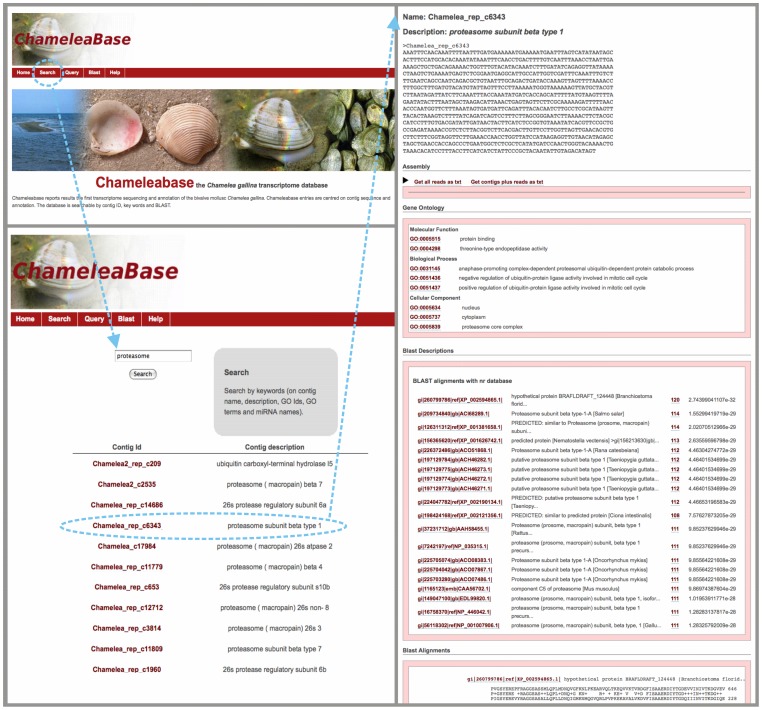
ChameleaBase. The screenshots report the *Chamelea gallina* database online version homepage (on the upper right side), the search facility (bottom right) and an example of the gene-like entry (on the left).

The database is organized following a hierarchical arrangement of information drawn from *C. gallina* transcriptome (Fig. 4). Each assembled contig is displayed as a gene-like entry. Each entry entails (i) a Contig information containing a FASTA sequence for each contig (identified by ChameleaBase ID) together with a preliminary description following Blast2GO or the best hit when available; (ii) the Assembly details summarized as a list of reads belonging to the contig. This information can also be downloaded in two FASTA files containing the contig, all read sequences and a multiple alignment between the contig and the reads; (iii) the principal BLAST output indicating, for both nucleotide and protein databases, results of similarity searches shown in a dedicated section in the classic BLAST format. This section includes the list of alignments descriptions and the pairwise alignments details with hits hyperlinked to external databases entries. Finally, the database reports the Gene Ontology search results describing each GO term associated to transcripts annotated with Blast2GO. The database is searchable by keywords and by BLAST, using nucleotide or protein sequences. It additionally implements a query system for massive data retrieval. For contigs, selected by GO terms ID or by keywords on contigs and BLAST hits descriptions, a customizable *.tsv file can be retrieved with data regarding contig ID, description and sequence as well as associated GO IDs and terms. Beside this information, FASTA and ACE files with reads/contigs alignments can be directly downloaded from the homepage. These features are extremely useful to facilitate large scale further bioinformatics analysis of the transcriptome, as well as for high throughput experiments design as custom microarray production and application and/or targeted re-sequencing.

## Conclusions

This study represents the first attempt to sequence and *de novo* annotate the transcriptome of the economically relevant species *C. gallina*. Given that very little about the genetics of this species is known, such knowledge will greatly improve the ability to manage genetic diversity in natural populations. In particular, the transcriptome information collected for this species holds the promise to shed light on different aspects of genetic mechanisms in several ways, underlying cellular and organism response to physiological and environmental stress. Nuclear markers, SSR and SNPs, polymorphism assessment will likely improve estimates of the effects of demographic processes, such as population declines and bottlenecks, effective population sizes, inbreeding levels and detection of minute levels of population structuring. It may additionally help to assign individuals of unknown origin to known baseline populations, for instance in mixed stock analyses [35]. Further possible applications in relation to conservation of this fishery resource would be the detection of local adaptation in order to understand the footprints of selection at the transcriptome level [35], [36]. Furthermore, these transcriptome resources could be searched for genes for a fine assessment of *C. gallina* expression patterns by custom microarray design. In this context, a careful selection of genes will be useful to address physiological and ecological questions involving for instance maturation, development, immune response, disease processes and host resistance, adaptation to changing environment conditions such as temperature increase or salinity fluctuation [14].

## Materials and Methods

### Biological samples and sequencing

This study did not involve experiments with live animals and has been conducted on invertebrates (molluscs) not subjected to regulations. No specific permits were required for the described field studies and *Chamelea gallina* samples were collected in July 2009 from Chioggia (between 0.3 and 3 miles away from the coast) from a commercial fisherman. No specific permissions were required for this location/activity, since the area is not privately-owned or protected and it is open to clam fishery. The fishing activity did not involve endangered or protected species.

Samples were immediately stored at −80°C to preserve genomic DNA and RNA for subsequent analysis. Total RNA from 4 adult individuals was extracted from 30 mg of muscle tissue of each individual using RNeasy mini-column kit (QIAGEN). Information on the sex of animals was not recorded. After checking the integrity, purity and size distribution of total RNA, samples were pooled and stored in three volumes of 96% ethanol and 0.1 volume of sodium acetate to obtain 5 µg of RNA in a final volume of 120 µl. Pooled RNA was sent to Evrogen (Moscow, Russia; http://www.evrogen.com) where double-stranded cDNA was synthesized using a SMART (Switching Mechanism At 5′ end of RNA Template) approach [37]. First-strand cDNA synthesis was performed with SMART Oligo II oligonucleotide (5′-AAGCAGTGGTATCAACGCAGAGTACGCrGrGrG-3′) and CDS-GSU primer (5′-AAGCAGTGGTATCAACGCAGAGTACCTGGAG-d(T)20-VN-3′) using 0.3 µg of total RNA. Double-strand cDNA was obtained from 1 µl of the first-strand reaction (5 times diluted with TE buffer) by PCR with SMART PCR primer (5′-AAGCAGTGGTATCAACGCAGAGT-3′). Amplified cDNA was purified using QIAquick PCR purification Kit (QIAGEN, CA). SMART prepared amplified cDNA was then normalized using the Duplex-Specific Nuclease (DSN) method [38]. In particular, normalization entailed cDNA denaturation/reassociation, DSN treatment and amplification of normalized fraction by PCR. SMART PCR primers were finally used to amplify 30 ng of normalized cDNA. Adapters were trimmed using GsuI (Fermentas) following the manufacturers protocol and cDNA purification was performed with Agencourt AMPure XP (BECKMAN COULTER). Approximately 15 µg of normalized cDNA were used for sequencing and library construction. Sequencing was performed at BMR Genomics, University of Padova, Italy (http://www.bmr-genomics.it) using one single region on a Genome Sequencer FLX instrument and GS FLX Titanium reagents. Bases were called with 454 Roche software by processing the pyroluminescence intensity for each bead-containing-well in each nucleotide incorporation. The software finally refines sequence information by removing adapter sequences.

### Transcriptome reconstruction

Raw reads, obtained from the sequencing, were first pre-processed by removing the PolyA tails and adaptors, using LUCY (http://lucy.sourceforge.net/) [39]. Moreover, all sequences smaller than 60 bases or with mean Phred quality lower than 30 were eliminated based on the assumptions that small reads might represent sequencing artifacts and low quality sequences might not be useful to the main data analysis.

Sequence reads were assembled into contigs by using the MIRA 3 assembler (Mimicking Intelligent Read Assembly; http://chevreux.org/projects_mira.html) [40]. Two runs of assembly were conducted by MIRA 3 in “EST” and “accurate” usage mode, respectively. Settings adopted for the two runs of ESTs were those defined by the 454 sequencing technology ([mira -project = chamelea_project -job = denovo,est,accurate,454 -notraceinfo 454_SETTINGS –CO: fnicpst = yes –CL: qc = no]).

### Annotation

*De novo* functional annotation of *C. gallina* transcriptome was obtained by similarity using BLAST, Blast2GO and custom made scripts. *De novo* functional annotation of the transcriptome was obtained by a multistep procedure, starting with BLAST similarity search (Basic Local Alignment Search Tool; ftp://ftp.ncbi.nlm.nih.gov/blast/db/) [41]. BLAST was run in local mode and assembled contigs were compared against the non-redundant (nr) protein database downloaded from BLAST site (release of October 4^th^ 2009, including all nr GenBank CDS translations+PDB+SwissProt+PIR+PRF) and to nt database. For both nr and nt searches, alignments with an e-value <1e−3 were retained.

The Blast2GO suite [42] was used for functional annotation of transcripts of *C. gallina* applying the function for the mapping of GO terms to transcripts with BLAST hits obtained from BLAST searches against nr. Only ontologies obtained from hits with e-value <1e−6, annotation cut-off >55, and a GO weight >5 were used for annotation.

CateGOrizer (previously known as “GO Terms Classifications Counter”; www.animalgenome.org/bioinfo/tools/countgo/); [43] was used to map GO terms to a restricted number of GO classes, for the three ontologies, and to count the number of occurrences of observed GO terms in functional classes.

### Polymorphic sequences detection: SSRs and SNPs

#### SSRs detection and *in vitro* validation

SSR motifs were identified using MISA 1.0 (MIcroSAtellite identification tool; http://pgrc.ipk-gatersleben.de/misa) [44], which identifies both perfect and compound repeats. Di-, tri-, tetra-, penta- and hexa-nucleotide repeats were searched for, with a minimum of six repeat units for dinucleotides, four for trinucletides and three repeat units for tetra, penta and hexanucleotides. Adjacent microsatellites ≤50 nt apart were considered compound repeats. Primer pairs were designed with FASTPCR 6.0 [45], following default program instructions. FASTPCR was also used to test for primer pairs compatibility to avoid primer dimers, self-annealing and hairpin formation when multiplexing loci during PCR. Primer validation was carried out on genomic DNA extracted from 12 *C. gallina* individuals (extraction protocol as in [46]) obtained from commercial fishermen out of Chioggia, in the same area as before, but in 2010.

Primers were tested in a PCR of 20 µl volume containing 1X reaction buffer (RBC Taq DNA Polymerase kit, RBC Bioscience), 0.07 mM dNTPs, 0.15 µM of each primer, 0.8 units Taq polymerase (5 units/µl, RBC Taq DNA Polymerase kit, RBC Bioscience) and 2 µl of genomic DNA (∼30 ng). PCR conditions were: initial denaturation at 94°C for 1 min., followed by 30 cycles of 94°C for 30 sec. (denaturation), 54–58°C for 30 sec. (annealing) (see Table 4 for detailed annealing temperature for each locus), 72°C for 30 sec. (extension) and a final single extension step at 72°C for 5 min. Electrophoresis was carried out at 100 V on 3% agarose TAE gels supplemented with 0.2 µg/ml of Gel Red (Biotium Inc.) for a preliminary polymorphism detection. Forward primers were labelled with FAM, VIC, NED and PET fluorescent dyes (Applied Biosystems) to verify the electrophoresis-predicted polymorphism. A fraction of the PCR product was loaded on an Applied Biosystems 3130 XL automated sequencer (Liz500 as size standard, genotyping facility at www.bmr-genomics.com) and allele sizes were assigned using GENEMARKER 1.71 (SoftGenetics, State College, Pennsylvania). Binning was automated with FLEXIBIN [47] and all input files for further analysis were produced with CREATE 1.33 [48]. Number of alleles and allele range were calculated with ARLEQUIN 3.5 [49]. Hardy-Weinberg equilibrium (Fisher's exact test) was tested with the software GENEPOP, online version [50] (nominal significant threshold α = 0.05). Null allele presence and frequency was detected with MICRO-CHECKER 2.2.3 [51].

#### SNPs detection

SNPs detection relies on two main steps: raw reads mapping to contigs obtaining quality-based multiple alignments, and alignment analysis to detect most probable single nucleotide polymorphic in the set of reads sequenced from different diploid individuals. SSAHA2 (Sequence Search and Alignment by Hashing Algorithm; http://www.sanger.ac.uk/resources/software/ssaha2/) [52] was used to re-map raw reads to contig sequences, to allow further SNPs discovery. FreeBayes [28], a Bayesian genetic variant detector, was used for SNP detection.

#### Pathogenic sequences detection

Using the NCBI Nucleotide Advanced Search Builder (http://www.ncbi.nlm.nih.gov/nuccore/advanced) all available nucleotide sequences pertaining to the taxonomic groups of Rhizaria, Alveolata (including Apicomplexa), and order Vibrionales were downloaded. Obtained fasta files were used to build local BLAST databases and the BLASTN program was used to query the databases with the *C. gallina* contigs as query sequences. Only matches longer than 100 nt with over 95% similarity and e-value smaller than 1e-3 were retained and taken into consideration. Only contigs with a significant match with pathogens sequences but without better matches in metazoans (according to previous BLAST search against nt) were reported.

## Supporting Information

Table S1 Putative infectious agents sequences found in *Chamelea gallina* transcriptome. The table reports best BLAST matches between *C. gallina* contig and the three pathogenic sequences databases analyzed. Results were filtered by highest Identity, then by best E-value and by longest Alignment length. Results were then validated by excluding matches, which included better Bit Scores with metazoans species. The last column reports species name corresponding to final best match.
(DOCX)

Table S2 Additional contigs annotated by searching the Pfam/Rfam databases. BLAST similarity search, run in local, was used to compare *Chamelea gallina* assembled contigs against Pfam protein families database and Rfam RNA families database. For both databases alignments with an e-value <1e−3 were retained. We obtained 6679 matches with Pfam and 30 matches with Rfam. The vast majority of these pointed to contigs that were already annotated. Only the 91 additional contigs, reported in the table, were newly annotated by BLAST search against Pfam.
(DOCX)
