# Supplementary material for: Sequencing and Characterization of Striped Venus Transcriptome Expand Resources for Clam Fishery Genetics
Source: PLoS One. 2012 Sep 18;7(9):e44185. doi: 10.1371/journal.pone.0044185 (PMC3445586; doi:10.1371/journal.pone.0044185)
Supplement: Table S1 — Putative infectious agents sequences found in Chamelea gallina transcriptome. The table reports best BLAST matches between C. gallina contig and the three pathogenic sequences databases analyzed. Results were filtered by highest Identity, then by best E-value and by longest Alignment length. Results were then validated by excluding matches, which included better Bit Scores with metazoans species. The last column reports species name corresponding to final best match. (DOCX) [file pone.0044185.s001.docx]

**Table S1 Putative infectious agents sequences found in *Chamelea gallina* transcriptome.** The table reports best BLAST matches between *C. gallina* contig and the three pathogenic sequences databases analyzed. Results were filtered by highest Identity, then by best E-value and by longest Alignment length. Results were then validated by excluding matches, which included better Bit Scores with metazoans species. The last column reports species name corresponding to final best match.

| **Query ID** | **Subject ID** | **Identity %** | **Alignment length** | **Mismatch** | **Gap open** | **Query start** | **Query end** | **Subject start** | **Subject end** | **E-value** | **Bit score** | **Species name** |
| --- | --- | --- | --- | --- | --- | --- | --- | --- | --- | --- | --- | --- |
| Chamelea_rep_c26848 | gi\|5348271\|gb\|AF094701.1\|AF0947 | 100 | 209 | 0 | 0 | 1 | 209 | 1054 | 846 | 9.00E-106 | 387 | *Vibrio sp.* |
| Chamelea_rep_c23211 | gi\|531550\|emb\|X76335.1\| | 100 | 172 | 0 | 0 | 29 | 200 | 879 | 1050 | 2.00E-85 | 318 | *Vibrio fluvialis* |
| Chamelea_rep_c19487 | gi\|56405805\|gb\|AY827492.1\| | 99.61 | 259 | 1 | 0 | 58 | 316 | 46 | 304 | 0 | 473 | *Vibrio olivaceus* |
| Chamelea_rep_c10306 | gi\|222436061\|gb\|ACFN01000029.1\| | 99.56 | 228 | 1 | 0 | 34 | 261 | 2569 | 2796 | 2.00E-114 | 416 | *Vibrio parahaemolyticus* |
| Chamelea_rep_c13602 | gi\|91187550\|gb\|AAPS01000068.1\| | 99.27 | 411 | 2 | 1 | 1 | 410 | 433 | 23 | 0 | 741 | *Vibrio alginolyticus* |
| Chamelea_rep_c18796 | gi\|9968810\|emb\|AJ294422.1\| | 99.07 | 216 | 1 | 1 | 32 | 246 | 930 | 1145 | 7.00E-106 | 387 | *Vibrio mediterranei* |
| Chamelea_rep_c4794 | gi\|22799135\|emb\|AJ316202.1\| | 98.91 | 733 | 7 | 1 | 32 | 764 | 173 | 904 | 0 | 1308 | *Vibrio sp.* |
| Chamelea_rep_c13766 | gi\|109729974\|gb\|CH902593.1\| | 98.88 | 626 | 5 | 2 | 13 | 637 | 294200 | 293576 | 0 | 1116 | *Vibrio alginolyticus* |
| Chamelea_c27157 | gi\|9968813\|emb\|AJ294425.1\| | 98.13 | 427 | 6 | 2 | 1 | 425 | 1837 | 1411 | 0 | 743 | *Vibrio splendidus* |
| Chamelea_rep_c14204 | gi\|37993648\|gb\|AY426981.1\| | 97.88 | 519 | 4 | 7 | 1 | 513 | 1276 | 759 | 0 | 893 | *Vibrio ezurae* |
| Chamelea_c36077 | gi\|91189679\|gb\|AAPS01000012.1\| | 97.37 | 304 | 6 | 2 | 24 | 326 | 102056 | 102358 | 1.00E-144 | 516 | *Vibrio alginolyticus* |
| Chamelea_rep_c29496 | gi\|222436061\|gb\|ACFN01000029.1\| | 96.13 | 388 | 8 | 7 | 34 | 417 | 2399 | 2783 | 7.00E-178 | 627 | *Vibrio parahaemolyticus* |
| Chamelea_c29838 | gi\|116070297\|gb\|AAKK02000057.1\| | 96.07 | 483 | 17 | 2 | 1 | 482 | 92 | 573 | 0 | 785 | *Vibrio sp.* |

Table headers: Query ID (*Chamelea gallina* contig identification number), Subject ID (Vibrionales sequence database identification number), Identity % (Percentage of identity between the query and the subject), Alignment length (number of aligned bp between query and subject), Mismatch, Gap open (number of gap opening in the alignment), Query start, Query end (first and last aligned base in the query sequence), Subject start, Subject end (first and last aligned base in the subject sequence), E-value (identity probability value), Bit score, Species name (species name corresponding to the Subject ID as reported in Genbank)
