# Supplementary material for: Sequencing and Characterization of Striped Venus Transcriptome Expand Resources for Clam Fishery Genetics
Source: PLoS One. 2012 Sep 18;7(9):e44185. doi: 10.1371/journal.pone.0044185 (PMC3445586; doi:10.1371/journal.pone.0044185)
Supplement: Table S2 — Additional contigs annotated by searching the Pfam/Rfam databases. BLAST similarity search, run in local, was used to compare Chamelea gallina assembled contigs against Pfam protein families database and Rfam RNA families database. For both databases alignments with an e-value <1e−3 were retained. We obtained 6679 matches with Pfam and 30 matches with Rfam. The vast majority of these pointed to contigs that were already annotated. Only the 91 additional contigs, reported in the table, were newly annotated by BLAST search against Pfam. (DOCX) [file pone.0044185.s002.docx]

**Table S2 Additional contigs annotated by searching the Pfam/Rfam databases.** BLAST similarity search, run in local, was used to compare *Chamelea gallina* assembled contigs against Pfam protein families database and Rfam RNA families database. For both databases alignments with an e-value <1e-3 were retained. We obtained 6679 matches with Pfam and 30 matches with Rfam. The vast majority of these pointed to contigs that were already annotated. Only the 91 additional contigs, reported in the table, were newly annotated by BLAST search against Pfam.

| **Query ID** | **Pfam ID** | **Family/Domain** |
| --- | --- | --- |
| Chamelea_rep_c22515 | PF05873.7 | Mt_ATP-synt_D |
| Chamelea_c17077 | PF00059.16 | Lectin_C |
| Chamelea_c20359 | PF07679.11 | I-set |
| Chamelea_c39909 | PF07690.11 | MFS_1 |
| Chamelea_c13151 | PF00059.16 | Lectin_C |
| Chamelea_rep_c34579 | PF00151.14 | Lipase |
| Chamelea_rep_c22720 | PF07679.11 | I-set |
| Chamelea_rep_c5921 | PF05903.9 | DUF862 |
| Chamelea2_c2911 | PF08190.7 | PIH1 |
| Chamelea_c20452 | PF07690.11 | MFS_1 |
| Chamelea_rep_c4911 | PF03645.8 | Tctex-1 |
| Chamelea_rep_c857 | PF00229.13 | TNF |
| Chamelea_c7813 | PF03803.10 | Scramblase |
| Chamelea_c17017 | PF08005.7 | PHR |
| Chamelea_rep_c528 | PF00059.16 | Lectin_C |
| Chamelea_c40148 | PF03372.18 | Exo_endo_phos |
| Chamelea2_rep_c2768 | PF05821.6 | NDUF_B8 |
| Chamelea_c38832 | PF07679.11 | I-set |
| Chamelea_rep_c346 | PF05821.6 | NDUF_B8 |
| Chamelea_rep_c11151 | PF05903.9 | DUF862 |
| Chamelea_rep_c40817 | PF00235.14 | Profilin |
| Chamelea_c7172 | PF12697.2 | Abhydrolase_6 |
| Chamelea_rep_c12263 | PF00582.21 | Usp |
| Chamelea_c22025 | PF00106.20 | adh_short |
| Chamelea_c17205 | PF01795.14 | Methyltransf_5 |
| Chamelea_rep_c12191 | PF00307.26 | CH |
| Chamelea_rep_c23876 | PF00708.13 | Acylphosphatase |
| Chamelea_c17351 | PF00001.16 | 7tm_1 |
| Chamelea2_c366 | PF01965.19 | DJ-1_PfpI |
| Chamelea2_c2407 | PF01569.16 | PAP2 |
| Chamelea_rep_c24501 | PF05903.9 | DUF862 |
| Chamelea_c22553 | PF00069.20 | Pkinase |
| Chamelea_c18940 | PF09747.4 | DUF2052 |
| Chamelea_rep_c2198 | PF03567.9 | Sulfotransfer_2 |
| Chamelea_c31787 | PF03281.9 | Mab-21 |
| Chamelea_c29393 | PF13902.1 | R3H-assoc |
| Chamelea_c9901 | PF05903.9 | DUF862 |
| Chamelea_c32300 | PF12657.2 | TFIIIC_delta |
| Chamelea_rep_c1329 | PF06140.8 | Ifi-6-16 |
| Chamelea_rep_c4761 | PF00389.25 | 2-Hacid_dh |
| Chamelea_rep_c1282 | PF08997.5 | UCR_6-4kD |
| Chamelea_rep_c30479 | PF08597.5 | eIF3_subunit |
| Chamelea_rep_c14287 | PF13695.1 | zf-3CxxC |
| Chamelea_c10858 | PF04145.10 | Ctr |
| Chamelea_c24587 | PF01926.18 | MMR_HSR1 |
| Chamelea_c18791 | PF00685.22 | Sulfotransfer_1 |
| Chamelea_c39839 | PF04194.8 | PDCD2_C |
| Chamelea_c26733 | PF00386.16 | C1q |
| Chamelea_rep_c22710 | PF00022.14 | Actin |
| Chamelea2_rep_c1347 | PF00229.13 | TNF |
| Chamelea_c18064 | PF07686.12 | V-set |
| Chamelea_rep_c30424 | PF06951.6 | PLA2G12 |
| Chamelea_c40220 | PF06784.6 | UPF0240 |
| Chamelea_c27584 | PF01569.16 | PAP2 |
| Chamelea_rep_c23263 | PF02820.13 | MBT |
| Chamelea_rep_c4085 | PF10574.4 | UPF0552 |
| Chamelea_c28536 | PF00012.15 | HSP70 |
| Chamelea_c17800 | PF01391.13 | Collagen |
| Chamelea_rep_c14193 | PF00687.16 | Ribosomal_L1 |
| Chamelea2_rep_c544 | PF00307.26 | CH |
| Chamelea_rep_c1926 | PF05873.7 | Mt_ATP-synt_D |
| Chamelea_c39741 | PF00135.23 | COesterase |
| Chamelea_rep_c8105 | PF05903.9 | DUF862 |
| Chamelea_c7227 | PF00386.16 | C1q |
| Chamelea_c27394 | PF06668.7 | ITI_HC_C |
| Chamelea_rep_c1897 | PF05821.6 | NDUF_B8 |
| Chamelea_rep_c28935 | PF00769.14 | ERM |
| Chamelea_c10832 | PF01965.19 | DJ-1_PfpI |
| Chamelea_c11445 | PF07690.11 | MFS_1 |
| Chamelea_c15243 | PF00059.16 | Lectin_C |
| Chamelea_rep_c41438 | PF05873.7 | Mt_ATP-synt_D |
| Chamelea_c25087 | PF13519.1 | VWA_2 |
| Chamelea_c10876 | PF09791.4 | Oxidored-like |
| Chamelea2_rep_c1823 | PF00389.25 | 2-Hacid_dh |
| Chamelea_rep_c12815 | PF04749.12 | PLAC8 |
| Chamelea_c37897 | PF00386.16 | C1q |
| Chamelea_c17953 | PF00188.21 | CAP |
| Chamelea_rep_c41351 | PF00889.14 | EF_TS |
| Chamelea_rep_c294 | PF08997.5 | UCR_6-4kD |
| Chamelea_rep_c17099 | PF00531.17 | Death |
| Chamelea_c40530 | PF13385.1 | Laminin_G_3 |
| Chamelea_rep_c20374 | PF00069.20 | Pkinase |
| Chamelea_c7391 | PF00932.14 | LTD |
| Chamelea_rep_c28966 | PF05721.8 | PhyH |
| Chamelea_c10867 | PF00078.22 | RVT_1 |
| Chamelea_c39687 | PF02932.11 | Neur_chan_memb |
| Chamelea_c16808 | PF01146.12 | Caveolin |
| Chamelea_c23782 | PF00001.16 | 7tm_1 |
| Chamelea_c38841 | PF03803.10 | Scramblase |
| Chamelea_c16197 | PF00024.21 | PAN_1 |
| Chamelea_rep_c5237 | PF01335.16 | DED |

Table headers: Query ID (*Chamelea gallina* contig identification number), Pfam ID (Protein families database identification number), Family/Domain (protein family or domain corresponding to each Pfam identification number).
